# Supplementary material for: Highways to happiness for autistic adults? Perceived causal relations among clinicians
Source: PLoS One. 2020 Dec 15;15(12):e0243298. doi: 10.1371/journal.pone.0243298 (PMC7737981; doi:10.1371/journal.pone.0243298)
Supplement: S1 Table — (DOCX) [file pone.0243298.s001.docx]

**S1 Table. List of abbreviations of nodes in the networks.**

| **Questionnaire** | **Abbreviation node** | **Content** |
| --- | --- | --- |
| Daily Life | adl | Problems with activities of daily life |
| (HONOS) | agr | Aggressive behavior |
|  | cog | Cognitive problems |
|  | com | Comorbid problems |
|  | drug | Problematic drug use |
|  | del | Problems due to delusions |
|  | dep | Problems due to depressive mood |
|  | livP | Problems with living situation |
|  | phyP | Physical problems |
|  | rel | Problems with relationships |
|  | shar | Self-mutilation |
| Well-being | coh | Satisfaction about cohabitants |
| (MANSA) | fam | Satisfaction about family relationships |
|  | fin | Satisfaction about financial situation |
|  | lei | Satisfaction about leisure time activities |
|  | lif | Life in general |
|  | liv | Satisfaction about living situation |
|  | nwor | Satisfaction about not working |
|  | onef | Having a (good) friend |
|  | phy | Satisfaction about physical health |
|  | psy | Satisfaction about psychological health |
|  | safe | Satisfaction about personal safety |
|  | sex | Satisfaction about sex life |
|  | ski | Opportunities to develop & use skills |
|  | soc | Satisfaction about quality of friendships |
|  | vis | Having met a (good) friend in the past week |
| Autism | inss | Subscale: Insistence on sameness |
| (ASBQ) | rcon | Subscale: Reduced contact |
|  | remp | Subscale: Reduced empathy |
|  | rsi | Subscale: Reduced social insight |
|  | ssms | Subscale: Sensory stimulation & motor stereotypies |
|  | vsc | Subscale: Violations of social conventions |
| General | comNR | No. of co-occurring diagnoses |
